# Minority species influences microbiota formation: the role of *Bifidobacterium* with extracellular glycosidases in bifidus flora formation in breastfed infant guts

**DOI:** 10.1111/1751-7915.13366

**Published:** 2019-01-13

**Authors:** Aina Gotoh, Miriam Nozomi Ojima, Takane Katayama

**Affiliations:** ^1^ Graduate School of Biostudies Kyoto University Sakyo‐ku Kyoto 606‐8502 Japan

## Abstract

The human body houses a variety of microbial ecosystems, such as the microbiotas on the skin, in the oral cavity and in the digestive tract. The gut microbiota is one such ecosystem that contains trillions of bacteria, and it is well established that it can significantly influence host health and diseases. With the advancement in bioinformatics tools, numerous comparative studies based on 16S ribosomal RNA (rRNA) gene sequences, metabolomics, pathological and epidemical analyses have revealed the correlative relationship between the abundance of certain taxa and disease states or amount of certain causative bioactive compounds. However, the 16S rRNA‐based taxonomic analyses using next‐generation sequencing (NGS) technology essentially detect only the majority species. Although the entire gut microbiome consists of 10^13^ microbial cells, NGS read counts are given in multiples of 10^6^, making it difficult to determine the diversity of the entire microbiota. Some recent studies have reported instances where certain minority species play a critical role in creating locally stable conditions for other species by stabilizing the fundamental microbiota, despite their low abundance. These minority species act as ‘keystone species’, which is a species whose effect on the community is disproportionately large compared to its relative abundance. One of the attributes of keystone species within the gut microbiota is its extensive enzymatic capacity for substrates that are rare or difficult to degrade for other species, such as dietary fibres or host‐derived complex glycans, like human milk oligosaccharides (HMOs). In this paper, we propose that more emphasis should be placed on minority taxa and their possible role as keystone species in gut microbiota studies by referring to our recent studies on HMO‐mediated microbiota formation in the infant gut.

## Introduction

The gut microbial composition, which significantly influences host health and diseases (Vijay‐Kumar *et al*., 2010; Kau *et al*., 2011; Kinross *et al*., 2011; Iida *et al*., 2013; Sommer and Bäckhed, 2013), changes over time, with the most drastic changes occurring at the onset and termination of breastfeeding (Yatsunenko *et al*., 2012). Bifidobacteria are the first colonizers in the intestines of breastfed infants. In many cases, bifidobacteria occupy more than 70% of the total infant gut microbiota (Tannock *et al*., 2013; Matsuki *et al*., 2016; Yamada *et al*., 2017). The lack of a bifidobacteria‐rich gut microbiota (bifidus flora) during infancy has been shown to be linked to a variety of health conditions (Brown *et al*., 1989; López‐Alarcón *et al*., 1997; von Kries *et al*., 1999; Olszak *et al*., 2012; Cox *et al*., 2014), including diarrhoea, allergy, atopic dermatitis, impaired immune responses and elevated serum cholesterol levels (Kalliomäki *et al*., 2001; Di Gioia *et al*., 2014) that continue throughout adulthood. Thus, a thorough understanding of the mechanisms that shape and modulate the infant bifidus flora within the gut microbiota is an important approach to address long‐term health problems.

The key modulator within breast milk is human milk oligosaccharides (HMOs). Despite being the third most abundant solid component in breast milk after lactose (Lac) and lipids, HMOs have no nutritional value for infants because of their resistance to pancreatic digestion (Kunz *et al*., 2000; Urashima *et al*., 2012). Several groups, including our own, have found the gene sets coding for enzymes that degrade HMOs (Sela *et al*., 2008; Garrido *et al*., 2016; James *et al*., 2016; Katayama, 2016; Matsuki *et al*., 2016) and have shown that these genes are limited to the infant gut‐associated bifidobacterial species among gut microbes (Ruiz‐Moyano *et al*., 2013; Katayama, 2016; Thomson *et al*., 2017). These findings suggest that it is highly likely that HMOs serve as selective nutrients for bifidobacterial species.

The bifidus flora mainly comprises four bifidobacterial species, *Bifidobacterium breve*,* Bifidobacterium bifidum*,* Bifidobacterium longum* subsp. *longum* (*B. longum*) and *Bifidobacterium longum* subsp. *infantis* (*B. infantis*), that show varied HMO assimilation phenotypes due to their different genotypes at the species and strain levels. Although the bifidus flora in the infant gut is a relatively simple microbial community compared to that of adults at the genus level, it is highly diverse and complex at the genotype level. Our previous report shows that within the bifidus flora, the minority species/strain act as potential keystone species that promote and maintain the bifidus flora.

**Figure 1:**
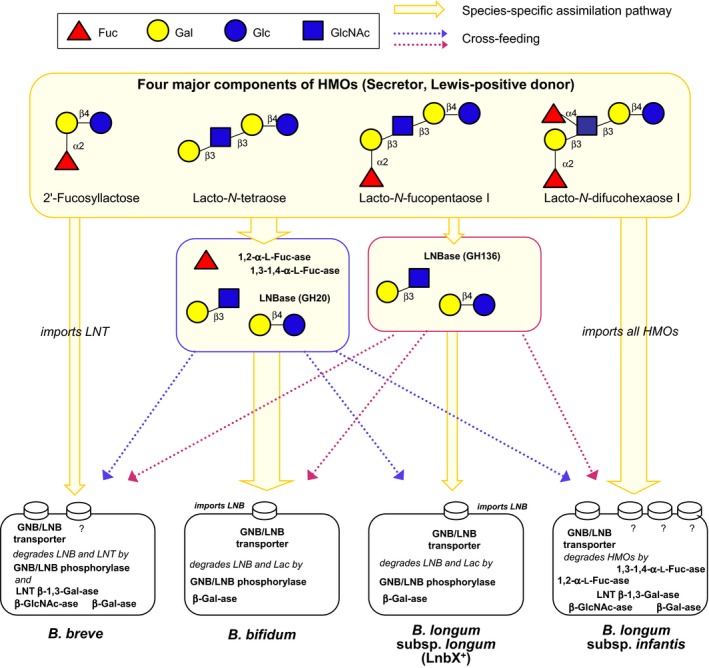
Structures of the four main human milk oligosaccharides (HMOs), and the assimilation pathways and enzymes utilized by each infant gut‐associated bifidobacterial species. Solid arrows indicate species‐specific assimilation pathways, and dotted arrows indicate potential cross‐feeding. *B. breve* and *B. longum* generally utilize LNT, while both *B. infantis* and *B. bifidum* consume a variety of HMOs. *B. bifidum* possesses cell surface‐attached enzymes that allow for extracellular degradation of HMOs. LNB ^+^−*B. longum* degrades LNT to LNB and Lac. Degradants produced and left unconsumed by *B. bifidum* and *B. longum* may be shared among the other bifidobacteria expressing both the GNB/LNB transporters and GNB/LNB phosphorylases. This figure was modified and adapted from the review article by Katayama (2016).

A keystone species is a species whose effect on the community is disproportionately large compared to its relative abundance (Paine, 1969; Power *et al*., 1996) and it alters their community through a variety of mechanisms. Most commonly cited examples, such as sea otters in kelp forests (Estes and Palmisano, 1974) and starfish in the rocky intertidal (Paine, 1966), are often apex predators. In the gut microbiota, however, keystone species primarily affect the community through altruistic degradation of substrates, which are otherwise recalcitrant, but are made available for other bacterial species to consume (Ze *et al*., 2012, 2013; Goodrich *et al*., 2014; Trosvik and de Muinck 2015; Centanni *et al*., 2018). One such recalcitrant substrate present in the infant gut ecosystem is HMOs. In human milk, HMOs with a variety of structures are included: lacto‐*N*‐tetraose (LNT: Galβ1‐3GlcNAcβ1‐3Galβ1‐4Glc) that contains lacto‐*N*‐biose I (LNB: Galβ1‐3GlcNAc) residue at the non‐reducing terminus of Lac, fucosylated LNT such as lacto‐*N*‐fucopentaose I (Fucα1‐2Galβ1‐3GlcNAcβ1‐3Galβ1‐4Glc) and lacto‐*N*‐difucohexaose I (Fucα1‐2Galβ1‐3(Fucα1‐4)GlcNAcβ1‐3Galβ1‐4Glc), and fucosylated Lac such as 2′‐fucosyllactose (Fucα1‐2Galβ1‐4Glc) and 3‐fucosyllactose (Galβ1‐4(Fucα1‐3)Glc; Fig. 1). Previously reported *in vitro* culture experiments showed that infant gut‐associated *Bifidobacterium* species have different HMO consumption behaviours (Fig. 1). For example, the fucosylated oligosaccharides were well assimilated by *B. infantis* and *B. bifidum*. However, the ability to utilize HMOs of *B. longum* and *B. breve* was restricted to LNT and LNB that is produced through LNT degradation (Asakuma *et al*., 2011), and only a limited number of *B. longum* and *B. breve* strains possess the enzyme set that degrades fucosylated HMOs (James *et al*., 2016; Matsuki *et al*., 2016). Despite their limited ability to degrade HMOs, *B. longum* and *B. breve* are frequently dominant species in the bifidus flora. This suggests that it is difficult to describe the mechanism of bifidus flora formation through *in vitro* HMO assimilation phenotype of each species and strain. Thus, we focused on the altruistic role (cross‐feeding) that minority species play within the gut microbiota.

## Case studies

### LnbX^+^−*B. longum*

A few rare strains of *B. longum* have the extracellular enzyme lacto‐*N*‐biosidase (LnbX), which degrades LNT to LNB and Lac (Fig. 1; Sakurama *et al*., 2013). LnbX can act not only on LNT, but also on human‐derived glycoconjugate sugars (Gotoh *et al*., 2015). We demonstrated that LnbX^+^−*B. longum* strains promote the growth of other bifidobacteria through cross‐feeding of LNT by‐products when co‐cultured in medium containing LNT as a carbon source. To assess the contribution of LnbX to the bifidus flora formation, we determined the prevalence of *lnbX* gene, *B. longum* (species level) and *Bifidobacterium* (genus level) in stools of both exclusively breastfed and mixed‐fed (formula‐ and breastfed) infants, by quantitative PCR (qPCR). As a result, we confirmed that the abundance of *Bifidobacterium* and *B. longum* in stools of breastfed infants was significantly higher than that of mixed‐fed infants. While *lnbX* expression was detected in five out of 10 (50%) individuals in the breastfed group, it was detected in only 17% of the individuals in the mixed‐fed group. In addition, we observed a positive correlation between the abundance of *lnbX* and *B. longum* in the stools of exclusively breastfed infants. On the other hand, no correlation was observed between the two factors for the stools of mixed‐fed infants. Interestingly, *B. longum* carrying the *lnbX* gene was, on average, only about 0.2% of the total *B. longum* population. These findings suggest that, although the LnbX^+^−*B. longum* strain is a minority species, it significantly contributes to the formation of bifidus flora through cross‐feeding of degraded by‐products of LNT (Yamada *et al*., 2017).

### *B. bifidum*

*Bifidobacterium bifidum*, which is generally known to be the minority species in the bifidus flora, expresses various glycosidases as cell surface‐anchored extracellular enzymes (Katayama *et al*., 2004; Wada *et al*., 2008; Ashida *et al*., 2009; Miwa *et al*., 2010) and has high viability when cultured with HMOs (Asakuma *et al*., 2011). Four strains of *B. bifidum* isolated from infant stools produced degradants of HMOs, such as LNB, Lac, fucose and galactose in culture supernatant during the logarithmic growth phase. This suggested that *B. bifidum* leaves the degraded mono‐ and oligosaccharides in the medium without using them immediately. When *B. longum* 105‐A strain, which assimilates only LNT, was co‐cultured with *B. bifidum* in the presence of HMOs, its growth was remarkably promoted (Gotoh *et al*., 2018). Stool samples collected from infants, children and adults were cultured in the presence or absence of each of the four strains of *B. bifidum* in the medium containing glucose (Glc) or HMOs. The total abundance of bacteria, *Bifidobacterium* (genus level) and *B. bifidum* (species level) was measured using qPCR. As a result, when the stool samples were cultured in the media supplemented with Glc, the abundance of *Bifidobacterium* either significantly decreased or did not change, compared to the group without the addition of *B. bifidum*. On the other hand, when cultured with HMOs, the exogenous addition of *B. bifidum* significantly increased the total abundance and prevalence of species in the *Bifidobacterium* genus other than *B. bifidum* (Table 1). This increase was strongly promoted in the cultured sample from an infant born through Caesarean section, in which the bifidus flora was not initially confirmed (Table 1, Infant C). We found that promoting the growth of preexisting *Bifidobacterium* was difficult when only HMOs were added, but the addition of *B. bifidum* stimulated the formation of the bifidus flora (Gotoh *et al*., 2018).

**Table 1:** Addition of *B. bifidum* to faecal suspensions incubated in the presence of HMOs enriches the *Bifidobacterium* population (species other than *B. bifidum*) in the culture. Prevalence was calculated by dividing total bifidobacterial 16S rRNA gene counts (except for *B. bifidum*) by total bacterial 16S rRNA gene counts. The data were adapted from the paper by Gotoh *et al*., 2018

*Bifidobacterium bifidum* strain added to faecal suspension	Faecal suspension (Age/Delivery mode)	Child A (4 years/vaginal)	Child B (5 years/vaginal)	Infant C (4 months/caesarean)	Adult D (30 years/no data)	Adult E (39 years/no data)
None added	Total bacteria (copies/ml; ×10^13^)	1.4 ± 0.5	1.7 ± 0.1	0.78 ± 0.01	1.7 ± 0.1	2.5 ± 0.0
	Prevalence of other bifidobacterial species in total bacteria (%)	0.0050	2.6	0.00034	0.37	5.0
JCM1254	Total bacteria (copies/ml×; ×0^13^)	2.0 ± 0.1	2.5 ± 0.4	1.7 ± 0.0	2.2 ± 0.1	2.4 ± 0.1
	Prevalence of other bifidobacterial species in total bacteria (%)	0.21	4.3	0.27	2.4	3.6
JCM7004	Total bacteria (copies/ml; × 10^13^)	1.6 ± 0.1	2.0 ± 0.2	1.3 ± 0.0	2.2 ± 0.1	2.6 ± 0.1
	Prevalence of other bifidobacterial species in total bacteria (%)	0.58	3.4	0.81	2.8	4.4
TMC3108	Total bacteria (copies/ml; ×10^13^)	2.2 ± 0.8	1.4 ± 0.1	1.1 ± 0.0	2.1 ± 0.1	2.7 ± 0.1
	Prevalence of other bifidobacterial species in total bacteria (%)	1.0	4.9	1.6	0.48	4.4
TMC3115	Total bacteria (copies/ml; ×10^13^)	1.3 ± 0.0	1.6 ± 0.1	1.4 ± 0.0	2.0 ± 0.1	2.5 ± 0.1
	Prevalence of other bifidobacterial species in total bacteria (%)	5.3	1.9	2.0	4.4	3.8

## Caveats

Although the two above‐mentioned studies indicated that minority species had a significant effect on microbiota formation, these studies have only examined a small snapshot of 24 h. To determine whether a species acts as a keystone species with confidence, future studies will need to examine the microbiota over a longer time period and perform a community time series analysis (Trosvik and de Muinck, 2015). Furthermore, the role of a keystone species is highly context‐dependent (Power *et al*., 1996). Keystone species may not always be the controlling agent at all times, but rather only under certain conditions. In the examples that we raised, the presence of an exclusive carbon source like HMOs allowed *B. bifidum* and LnbX^+^−*B. longum* to act as a keystone species in the bifidus flora (Yamada *et al*., 2017; Gotoh *et al*., 2018).

## Conclusions

In the adult intestine, members of the gut microbiome exhibit complex cross‐feeding. Previous reports show several examples of microbe–microbe relationships, such as bidirectional feeding between *Bacteroides ovatus* and *Bacteroides vulgatus*, which is mediated through inulin (Rakoff‐Nahoum *et al*., 2016), and between *Akkermansia muciniphila* and *Eubacterium hallii*, which is mediated through *O*‐glycan degradants derived from mucin and pseudovitamin B12 (Belzer *et al*., 2017). Unidirectional feeding was also observed between *A. muciniphila* and *Anaerostipes caccae*, and between *B. adolescentis* and *Faecalibacterium prausnitzii* (Rios‐Covian *et al*., 2015). These examples show that Actinobacteria, the phylum to which *Bifidobacterium* belongs, and Verrucomicrobia, to which *Akkermansia* belongs, are minority phyla in human gut microbiota that influence the abundance of species that belong to other phyla, such as Bacteroidetes and Firmicutes.

In the infant gut, the most abundant genus is generally *Bifidobacterium*, and the carbon source that is the most available to them comes from HMOs in breast milk. Interestingly, the four infant gut‐associated bifidobacterial species and their multiple strains have evolved different strategies to degrade HMOs and to maintain diversity. We demonstrated that *B. longum* strains that express *lnbX* and *B. bifidum* are potential keystone species in the establishment of the bifidus flora by providing HMO degradants for other bacterial groups to use. In other words, the cross‐feeding between minority taxa and dominant taxa is an important mechanism for the formation and maintenance of a diverse bifidus flora (Fig. 1). These findings enhance our understanding of how the bifidus flora is formed and, by conducting follow‐up microbiota studies of different individuals, can provide insight into how the physiology and ecology of the gut microbiota potentially affect human health.

## Conflict of interest

None declared.
